# Supplementary material for: Comparative genomics provides new insights into the diversity, physiology, and sexuality of the only industrially exploited tremellomycete: Phaffia rhodozyma
Source: BMC Genomics. 2016 Nov 9;17:901. doi: 10.1186/s12864-016-3244-7 (PMC5103461; doi:10.1186/s12864-016-3244-7)
Supplement: Additional file 6: — List of orphan genes with links to PFAM (related to Additional file 1: Table S1). (ZIP 1428 kb) [file 12864_2016_3244_MOESM6_ESM.zip › PFAM_UNIQUE/G02198_P.html]

#                                                                            --- full sequence --- -------------- this domain -------------   hmm coord   ali coord   env coord
  
# target name        accession   tlen query name           accession   qlen   E-value  score  bias   #  of  c-Evalue  i-Evalue  score  bias  from    to  from    to  from    to  acc description of target
  
#------------------- ---------- ----- -------------------- ---------- ----- --------- ------ ----- --- --- --------- --------- ------ ----- ----- ----- ----- ----- ----- ----- ---- ---------------------
  
G02198\_P             -            217 Defensin\_propep      PF00879.13    52       3.2    6.5   7.3   1   2     0.016        96    1.7   0.6    33    48    40    55    26    58 0.71 - Defensin propeptide  
G02198\_P             -            217 Defensin\_propep      PF00879.13    52       3.2    6.5   7.3   2   2   0.00023       1.4    7.7   1.1    20    49   126   155   117   158 0.88 - Defensin propeptide  
G02198\_P             -            217 ERM                  PF00769.14   246       2.5    6.2   9.6   1   2       0.6        88    1.2   0.1   131   194    17    80     5    84 0.42 - Ezrin/radixin/moesin family  
G02198\_P             -            217 ERM                  PF00769.14   246       2.5    6.2   9.6   2   2     0.012       1.8    6.7   5.7    99   172    86   162    81   166 0.87 - Ezrin/radixin/moesin family  
G02198\_P             -            217 PBP1\_TM              PF14812.1     81       2.2    7.3   6.7   1   2      0.29        45    3.1   1.9    35    53    31    49    21    66 0.60 - Transmembrane domain of transglycosylase PBP1 at N-terminal  
G02198\_P             -            217 PBP1\_TM              PF14812.1     81       2.2    7.3   6.7   2   2     0.024       3.8    6.6   0.2    21    62   118   158   108   173 0.50 - Transmembrane domain of transglycosylase PBP1 at N-terminal
